# Genome-wide association mapping for resistance to leaf, stem, and yellow rusts of common wheat under field conditions of South Kazakhstan

**DOI:** 10.7717/peerj.9820

**Published:** 2020-08-31

**Authors:** Yuliya Genievskaya, Yerlan Turuspekov, Aralbek Rsaliyev, Saule Abugalieva

**Affiliations:** 1 Plant Molecular Genetics Laboratory, Institute of Plant Biology and Biotechnology, Almaty, Kazakhstan; 2 Biodiversity and Bioresources, Al-Farabi Kazakh National University, Almaty, Kazakhstan; 3 Laboratory of Phytosanitary Safety, Research Institute of Biological Safety Problems, Gvardeisky, Zhambyl Region, Kazakhstan; 4 Kazakh National Agrarian University, Almaty, Kazakhstan

**Keywords:** Common wheat, Association mapping, Leaf rust, Stem rust, Yellow rust, Quantitative trait loci, Single nucleotide polymorphism

## Abstract

Common or bread wheat (*Triticum aestivum* L.) is the most important cereal crop in the world, including Kazakhstan, where it is a major agricultural commodity. Fungal pathogens producing leaf, stem, and yellow (stripe) rusts of wheat may cause yield losses of up to 50–60%. One of the most effective methods for preventing these losses is to develop resistant cultivars with high yield potential. This goal can be achieved using complex breeding studies, including the identification of key genetic factors controlling rust disease resistance. In this study, a panel consisting of 215 common wheat cultivars and breeding lines from Kazakhstan, Russia, Europe, USA, Canada, Mexico, and Australia, with a wide range of resistance to leaf rust (LR), stem rust (SR), and yellow rust (YR) diseases, was analyzed under field conditions in Southern Kazakhstan. The collection was genotyped using the 20K Illumina iSelect DNA array, where 11,510 informative single-nucleotide polymorphism markers were selected for further genome-wide association study (GWAS). Evaluation of the phenotypic diversity over 2 years showed a mostly mixed reaction to LR, mixed reaction/moderate susceptibility to SR, and moderate resistance to YR among wheat accessions from Kazakhstan. GWAS revealed 45 marker–trait associations (MTAs), including 23 for LR, 14 for SR, and eight for YR resistances. Three MTAs for LR resistance and one for SR resistance appeared to be novel. The MTAs identified in this work can be used for marker-assisted selection of common wheat in Kazakhstan in breeding new cultivars resistant to LR, SR, and YR diseases. These findings can be helpful for pyramiding genes with favorable alleles in promising cultivars and lines.

## Introduction

Wheat is one of the most economically important agricultural crops in the world, including in Kazakhstan, where its sowing area occupies 78.1% of all cereal fields (Statistics Committee, 2019). Kazakhstan produces 20–25 million tons of common wheat per year, and exports up 5–7 million tons of the grain. Rust diseases are largest threats to wheat production in Kazakhstan.

Among wheat rust diseases, leaf rust (LR), caused by *Puccinia recondita* Rob. ex Desm f. sp. *Tritici*, is the most common for many wheat-producing areas around the world, where it may cause substantial yield losses (Marasas, Smale & Singh, 2004) due to reduced kernel number and weight. Occasionally, LR causes serious yield losses of spring wheat in Kazakhstan, especially in northern Kazakhstan, where spring wheat prevails. For example, in 2000–2001 in the Akmola region, epiphytotic development of this disease resulted in 50–100% loss in productivity (Koyshybaev, 2018). Approximately 80 LR resistance genes (*Lr*) have been identified in common and durum wheat and their diploid relatives (Qureshi et al., 2018). In Kazakhstan, research work has been ongoing over the past 10 years to identify *Lr* genes based on the screening of wheat cultivars for the presence of known resistance genes (Akhmetova et al., 2015; Rsymbetov et al., 2018) as well as investigations into a population of *P. recondita* f. sp. *tritici* in both the country and neighboring territories (Agabaeva & Rsaliyev, 2013; Gultyaeva et al., 2018).

Almost all commercial spring wheat cultivars grown in Kazakhstan show poor resistance to the stem rust (SR) pathogen *Puccinia graminis* Pers. f. sp. *tritici* Erikss. & Henn. (Marasas, Smale & Singh, 2004). Epiphytotic periods of this disease in Kazakhstan resulted in crop losses ranged from 40% to 60% (Koyshybaev, 2018). Therefore, the prevention of SR is an important issue, requiring comprehensive genetic and breeding studies. Several experiments were conducted in Kazakhstan to search for sources of SR resistance in wheat germplasm (Kokhmetova & Atishova, 2012; Shamanin et al., 2016; Rsaliyev & Rsaliyev, 2018). Nearly 60 *Sr* genes have been identified and cloned in wheat and its wild relatives (Chen et al., 2018). The highly virulent race Ug99 (*Sr31*) is not found in Kazakhstan. However, its area is continually widening, posing a future threat to the food security of the entire planet (Schumann & Leonard, 2000).

Yellow (or stripe) rust (YR) (*Puccinia striiformis* Westend.) infection is another serious threat to food security in many countries around the world. In Kazakhstan, YR is common in the foothill and mountain zones of the south and southeast, the main winter wheat growing regions (Koyshybaev, 2018). Epiphytotic development of YR caused by an abnormal amount of precipitation leads to severe crop reduction. For example, in 2002, it caused a 30–40% yield loss in the southeast region (Koyshybaev, 2018). About 70 *Yr* genes have been described in wheat, while many reported YR resistance genes need to be named (Waqar et al., 2018).

The genetic background of wheat resistance to the above-listed rust diseases is complex, due to the quantitative nature of the traits (Ellis et al., 2014), which is additionally complicated by the variability of pathogen races in certain environments (Marasas, Smale & Singh, 2004; Rsaliyev et al., 2005). Genomics-assisted breeding for disease resistance typically involves the following steps: gene identification, isolation, cloning, functional characterization to elucidate the genetic mechanisms of resistance, validation, and deployment (Juliana et al., 2018). The search for additional sources of resistance to rust diseases can be conducted using extensive germplasm collections representing different genetic backgrounds and origins (Kokhmetova et al., 2011; Badoni et al., 2017; Kumar et al., 2019). Linkage mapping has been commonly used to identify QTLs associated with resistance to SR (Wang et al., 2015; Chen et al., 2018), LR (Xue et al., 2018; Kthiri et al., 2018), and YR (Zeng et al., 2019). This method was also successfully applied in QTL mapping for LR and SR resistance in Kazakhstan (Genievskaya et al., 2019). However, linkage mapping has certain disadvantages, the most substantial of which is low allele richness and prolonged identification of resistant QTLs due to the long time required for the construction of a mapping population as well as the large sample sizes (Beavis, 1998; Xu et al., 2017).

The other approach is a natural population-based mapping that relies on the availability of diverse germplasms, large-scale phenotypic scoring, high allelic richness, and high-resolution genotyping (Korte & Farlow, 2013). Genome-wide association study (GWAS) has recently become commonly used for this type of QTL mapping. GWAS, as an efficient instrument, has been broadly used in many QTL mapping experiments associated with rust disease resistance in common wheat around the globe (Zegeye et al., 2014; Bajgain et al., 2015; Maccaferri et al., 2015). Until recently, the genetic aspects of wheat disease resistance based on GWAS have not yet been studied in Kazakhstan. However, a large collection of local accessions genotyped by 90K single-nucleotide polymorphism (SNP) Illumina array (Turuspekov et al., 2015) and 194 accessions harvested in southern, central, and northern regions of the country were used for the identification of marker-trait associations for yield components based on GWAS (Turuspekov et al., 2017). The purpose of this study was to identify QTLs for LR, SR, and YR resistance using GWAS in common wheat grown in Kazakhstan. The collection was studied for the presence of possible sources of resistance for wheat breeding.

## Materials and Methods

### Plant material for genotyping and phenotyping

A collection of 215 common wheat cultivars and breeding lines with a wide range of resistance to three rust diseases (leaf, stem, and yellow rust) was used for this study (Table S1). The germplasm set included (1) 91 commercial and prospective breeding cultivars of Kazakhstan and Russia, including 64 cultivars approved by the State Seed Trials Commission for use in the territory of Kazakhstan; (2) 38 cultivars from Europe received from the John Innes Centre, United Kingdom; and (3) 86 cultivars and lines from Kazakhstan, Russia, USA, Canada, Mexico, Germany, and Australia provided by the Research Institute of Biological Safety Problems (RIBSP, Gvardeisky, South Kazakhstan).

### Evaluation of LR, SR, and YR in the field

The three wheat rust diseases were studied in the infectious experimental fields of the RIBSP in 2018 and 2019. Each cultivar or line in the collection was sown on 0.4 m^2^ plots with two rows and a distance of 20 cm between rows. Plots were grown in randomized complete blocks in two independent replications. Each individual row was 100 cm long with 50–60 seeds per row. “Akmola 2” and “Astana” were used as check cultivars. For the accumulation and spreading of infection in the nursery, two susceptible cultivars, “Morocco” and “Saratovskaya 29”, were grown between experimental plots. The phenotypic data reported here are the average values of two independent replications. All wheat accessions were evaluated for resistance to bulked races of LR and SR obtained from commercial cultivars of common spring wheat grown in eastern, northern (Kostanay and Akmola regions), southeastern (Almaty region), and southern (Zhambyl region) Kazakhstan in 2016 and 2017, and stored in the collection of microorganisms at the RIBSP. Urediniospores of *P. striiformis* (YR) that were used as inoculum were obtained in the southern and southeastern parts of the country in 2016 and 2017. Plants were inoculated with spores of LR, SR, and YR at the booting stage.

The response of plants to pathogens was assessed at the seed ripening stage (70 on the Zadoks scale) for LR and YR, and at the milk development stage (75 on the Zadoks scale) for SR (Zadoks, Chang & Konzak, 1974). Plant resistance was determined by two indicators: the plant response to infection (the type of reaction) and the severity (the percentage of affected leaf area). The type of rust infection was determined according to the scale of Stakman, Stewart & Loegering (1962) for SR, the scale of Mains & Jackson (1926) for LR, and the scale of Gassner & Straib (1929) for YR. The severity of rust infection on leaf and stem surfaces was assessed using the modified Cobb scale (Peterson, Campbell & Hannah, 1948; Roelfs, Singh & Saari, 1992). To meet the data format requirements for association analysis, the conventional scale was converted to the 0–9 linear disease resistance scale described by Zhang, Bowden & Bai (2011).

### DNA extraction and SNP genotyping

Genomic DNA samples were extracted and purified from a single seedling of each individual cultivar or line using the cetyltrimethylammonium bromide method (Doyle & Doyle, 1990). The DNA concentration for each sample was adjusted to 50 ng/μL. All 215 accessions were genotyped using a 20K Illumina iSelect SNP assay at the TraitGenetics Company (TraitGenetics GmbH, Gatersleben, Germany). A total of 12,419 raw SNP markers were processed using the criteria for GWAS described by Miyagawa et al. (2008). According to these criteria, markers with call rate ≥90%, Hardy–Weinberg equilibrium fit at *P* ≥ 0.001, a confidence score of 0.5, and minor allele frequency (MAF) ≥5% were considered to meet the requirements. Accessions with greater than 15% missing data were also removed.

### Analysis of linkage disequilibrium, structure, kinship, and statistics in the studied population

The pairwise linkage disequilibrium (LD) between the markers based on their correlations (*R*^2^) was calculated using the Java-based open source software TASSEL v.5.2.53 (Bradbury et al., 2007). R statistical software was used to plot the correlation between pairwise *R*^2^ and the genetic distance (LD decay plot) (R Core Team, 2018). Population structure (Q) was analyzed using a model-based clustering method (admixture models with correlated allele frequencies) in STRUCTURE v.2.3.4 (Pritchard, Stephens & Donnelly, 2000). Five independent runs were conducted for each specified *K* (from 2 to 10), with a 100,000 burn-in length and 100,000 Markov chain Monte Carlo iterations. The optimal number of clusters (*K*) was chosen based on the *ΔK* method (Evanno, Regnaut & Goudet, 2005). The analysis was performed using a web-based tool called Structure Harvester v.0.6.94 (Earl & Von Holdt, 2012). TASSEL was also used to construct a population kinship matrix based on the scaled identity by state method and using the complete set of markers that met all requirements. ANOVA, Pearson correlation, and other descriptive statistics were analyzed using R statistical software (R Core Team, 2018). The broad-sense heritability index (*h_b_*^2^), describing the proportion of phenotypic variation due to genetic factors, was calculated based on the ANOVA outcome using

$${h}_{b}^2 = {\rm \; }\displaystyle{{{\rm S}{{\rm S}_{\rm g}}} \over {{\rm S}{{\rm S}_{\rm t}}}}$$

where SS_g_ is the sum of squares for genotype and SS_t_ is the total sum of squares.

### Association analysis and MTA mapping

To detect significant associations, the combined MLM + kinship matrix + Q matrix model (Yu et al., 2006) was applied using the TASSEL software. To confirm the correction due to K and Q matrices, the distribution lines in each quantile–quantile (QQ) plot were analyzed. Significant MTAs were selected after the application of a threshold at *P* < 1E−03. Positions and sequences of SNP markers were obtained from the 90K Array Consensus map of the common wheat genome (Wang et al., 2014). For markers with unknown positions in the 90K Array Consensus map, the CSS POPSEQ 2014 map (Edae, Bowden & Poland, 2015), available at the Triticeae Toolbox (2020), was used. In the case of several significant MTAs positioned closely to each other, the SNP with the lowest *P*-value was chosen. For the search of protein-coding genes that overlap with identified significant MTAs, the sequence of each marker was inserted into the BLAST tool (2020) of Ensembl Plants (2020) and compared with the reference genome of *T. aestivum*. Approximate positions of *Lr*, *Sr*, and *Yr* genes were obtained from several sources. For *Sr* genes, we used a consensus map for Ug99 SR resistance (Yu et al., 2014), and the Wheat-Composite2004 map from the GrainGenes database (GrainGenes, 2020). For *Lr* genes, we used genetic maps described by Terracciano et al. (2013), Aoun et al. (2016) and the Wheat-Composite2004 map. For *Yr* genes, we used a genetic map described by Maccaferri et al. (2015). MapChart v.2.32 software (Voorrips, 2002) was used to construct a genetic map with the significant MTAs and *Lr*, *Sr*, and *Yr* genes.

## Results

### Infection types of accessions to LR, SR, and YR in the field

**Figure 1:**
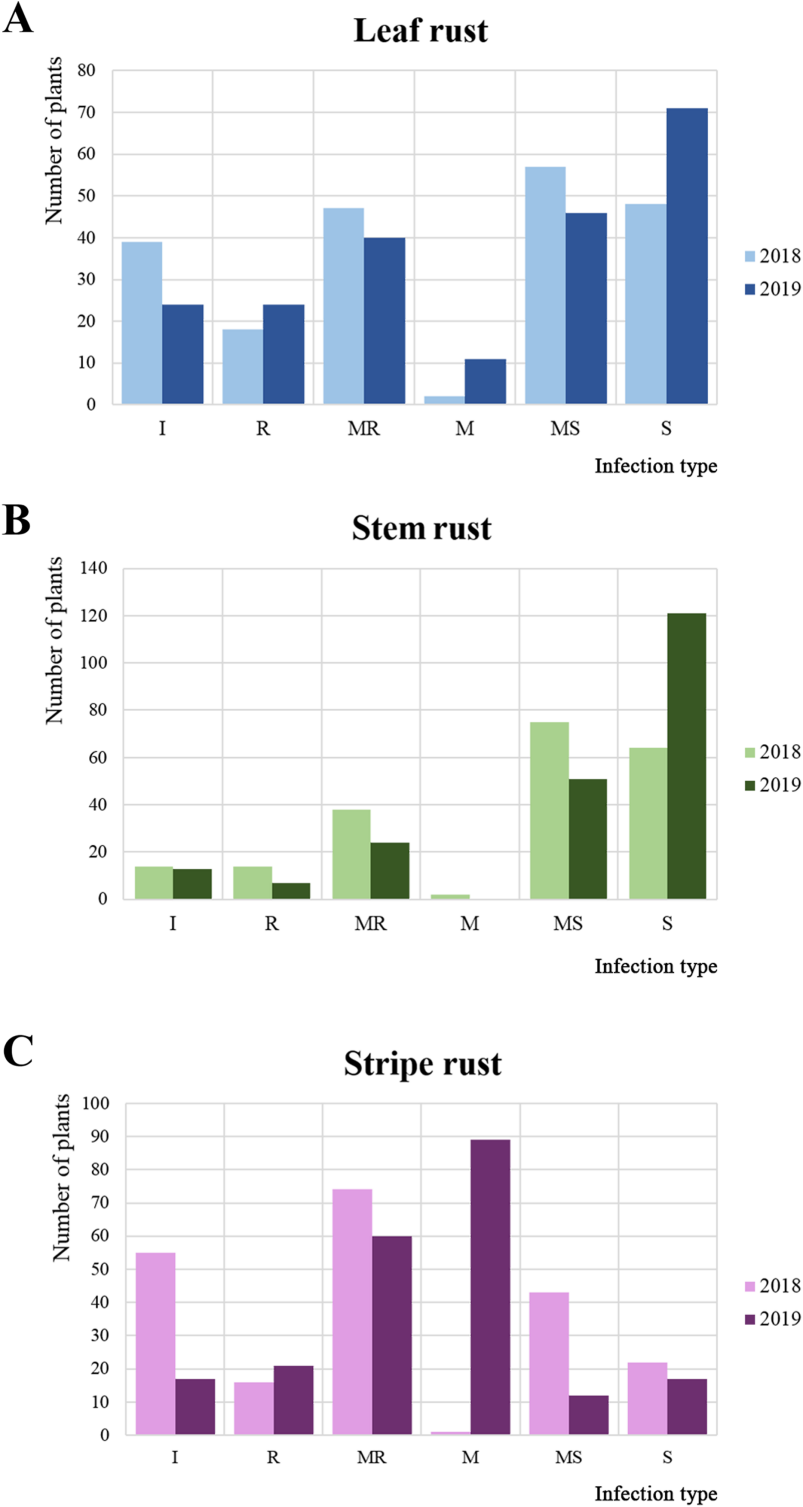
Variations in the reaction of wheat accessions to leaf, stem, and yellow rusts. Frequencies of each reaction type to (A) leaf rust (LR), (B) stem rust (SR), and (C) yellow rust (YR) as measured in 2 years, where I, R, MR, M, MS, and S represent immune, resistant, moderately resistant, mixed reaction, moderately susceptible, and susceptible reaction, respectively.

In 2018 and 2019, 215 common wheat accessions were evaluated in the field plots of the RIBSP for resistance to LR, SR, and YR (Fig. 1; Data S1). The mean LR score in 2018 was 4.4 ± 3.1 on a 0–9 scale. In 2018, about half of the collection was defined as moderately susceptible/susceptible (Fig. 1A). In 2019, the average LR score increased up to 4.7 ± 3.2.

For SR, the average infection type score in 2018 was 5.5 ± 2.4 (mixed/moderately susceptible), with the majority of the collection (67.1%) rated as moderately susceptible/susceptible (Fig. 1B). In 2019, the result was notably different from those in 2018. The percentage of moderately susceptible/susceptible increased to 79.6% (Fig. 1B). The average SR infection type score increased to 6.7 ± 3.0 (moderately susceptible) in 2019.

For YR, in 2018, the majority of accessions (42.7%) were resistant/moderately resistant (Fig. 1C). However, in 2019, the majority of the collection demonstrated a mixed reaction to YR (Fig. 1C). The average level of YR infection type score was stable at 3.2 ± 2.8 in 2018 and 3.4 ± 2.2 in 2019.

Pearson correlation analysis revealed a strong positive correlation between LR and SR in 2018 and 2019, and between SR and YR severities in 2018 (Table 1). A moderate positive correlation was observed between LR and YR in 2018, and a less strong but still significantly positive correlation between LR and YR and between SR and YR in 2019.

**Table 1:** Pairwise correlation coefficients (*r*) for the severities of three rust diseases in 2 years.

	2018			2019			
	LR	SR	YR	LR	SR	YR	
2018	LR	–					
	SR	0.494***	–				
	YR	0.217**	0.460***	–			
2019	LR	0.718***	0.393***	0.153*	–		
	SR	0.461***	0.603***	0.240***	0.504***	–	
	YR	0.305***	0.211**	0.318***	0.154*	0.136*	–

**Notes:**

* *P* < 0.05.

** *P* < 0.01.

*** *P* < 0.001.

LR, leaf rust; SR, stem rust.

The ANOVA test demonstrated strong genetic effect for all three diseases, whereas the effects of environment (E) and genotype × environment (G × E) were relatively lower (Table 2). The highest *h_b_*^2^ was observed for resistance to LR, and the lowest for resistance to YR.

**Table 2:** Summary of ANOVA analyses on leaf, stem, and yellow rusts severities and the estimated broad-sense heritability (*h_b_*^2^).

Factor	df	SS	MS	*F*	*h_b_*^2^
LR					
G	210	3,834.030	18.260	4.482***	0.857
E	1	11.046	10.960	2.045***	
G × E	210	630.000	3.000	1.139**	
Residuals			2.480		
SR					
G	210	2,867.410	13.650	2.460***	0.771
E	1	138.820	138.780	20.646**	
G × E	210	712.201	3.390	1.002**	
Residuals			1.086		
YR					
G	210	1,749.012	8.328	3.120**	0.652
E	1	6.400	6.408	2.895*	
G × E	210	926.600	4.412	1.985*	
Residuals			3.001		

**Notes:**

* *P* < 0.05.

** *P* < 0.01.

*** *P* < 0.001.

LR, leaf rust; SR, stem rust; YR, yellow rust, G, genotype; E, environment; G × E, genotype × environment; df, degree of freedom; SS, sum of squares; MS, mean squares; *h_b_*^2^, broad-sense heritability index.

### Genotyping results, linkage disequilibrium, and analysis of the population structure

After processing of genotyping data, 11,510 polymorphic SNP markers for 215 wheat accessions were selected for the GWAS (Data S2). Distribution of SNP markers among genomes was as follows: 2,186 for the A genome, 2,955 for the B genome, and 414 for the D genome. The remaining 5,955 markers had unknown genomic positions in the 20K array. Chromosome 2B had the largest number of markers (640 SNP) with chromosome 7A being the longest chromosome (216.0 cM). There were 1.6 markers/cM, on average, for the three genomes. The highest density was observed for genome B, with an average distance of 0.3 cM between markers. Generally, the density of the D genome was nearly seven times less than those of genomes A and B.

**Figure 2:**
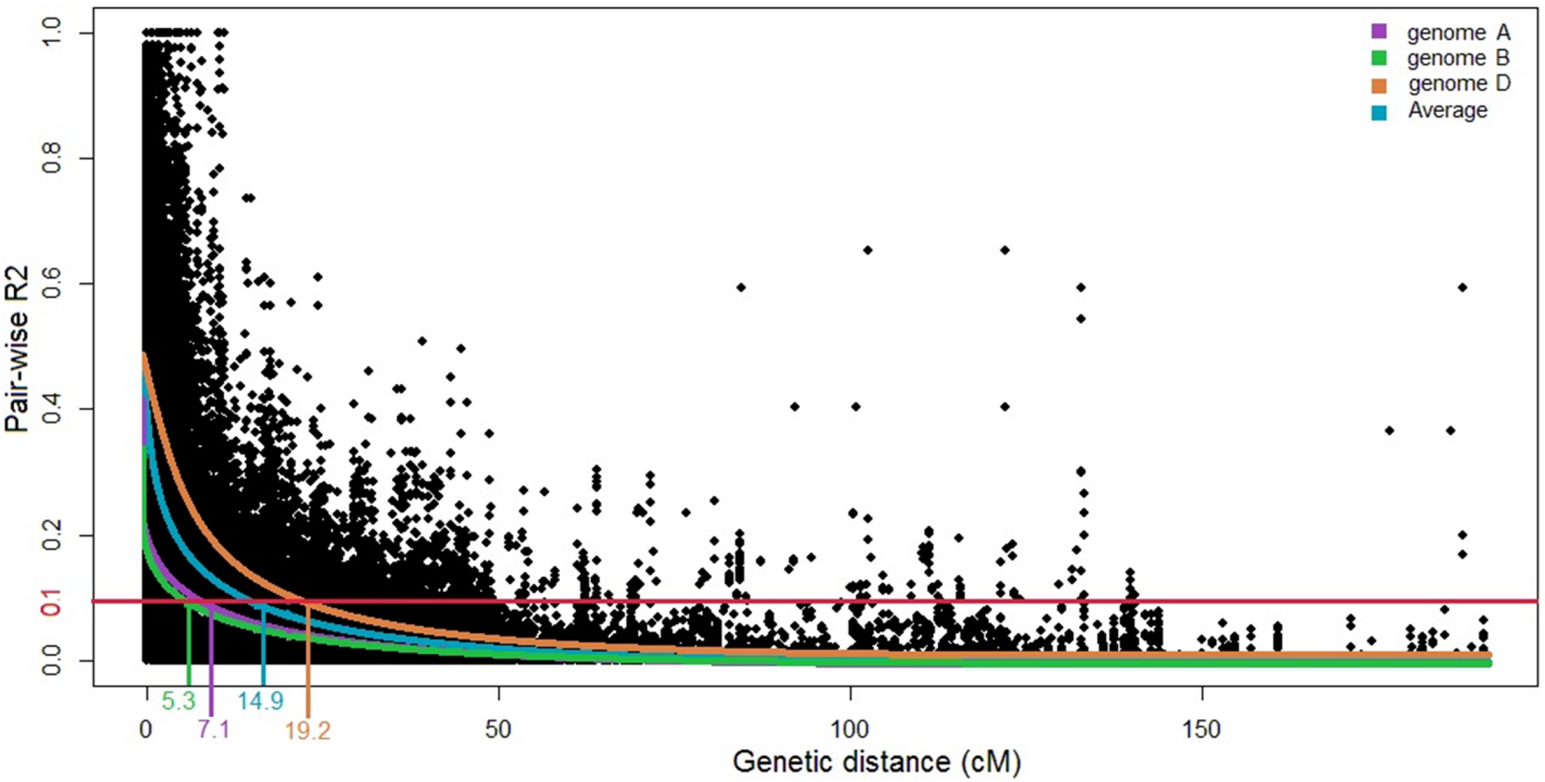
Scatter plot of linkage disequilibrium (LD) decays across the genomes and the average for three wheat genomes. The distance in centimorgans (cM) is plotted against the LD estimate (*R*^2^) for pairs of markers. The horizontal red line denotes the LD threshold of 0.1 for pair-wise *R*^2^.

Linkage disequilibrium decays at 14.9 cM for the whole genome at *R*^2^ of 0.1 (Fig. 2). Here, the LD decay at 7.1 and 5.3 cM in the A and B genomes, respectively; for the D genome, the LD extends to 19.2 cM. Based on the results of STRUCTURE and STRUCTURE Harvester analyses, the Q matrix was developed using *K* = 3 as the optimum. The generated Q matrix was used as a covariate matrix for MLM in TASSEL.

### Putative novel MTAs identified in comparison to existing references

**Figure 3:**
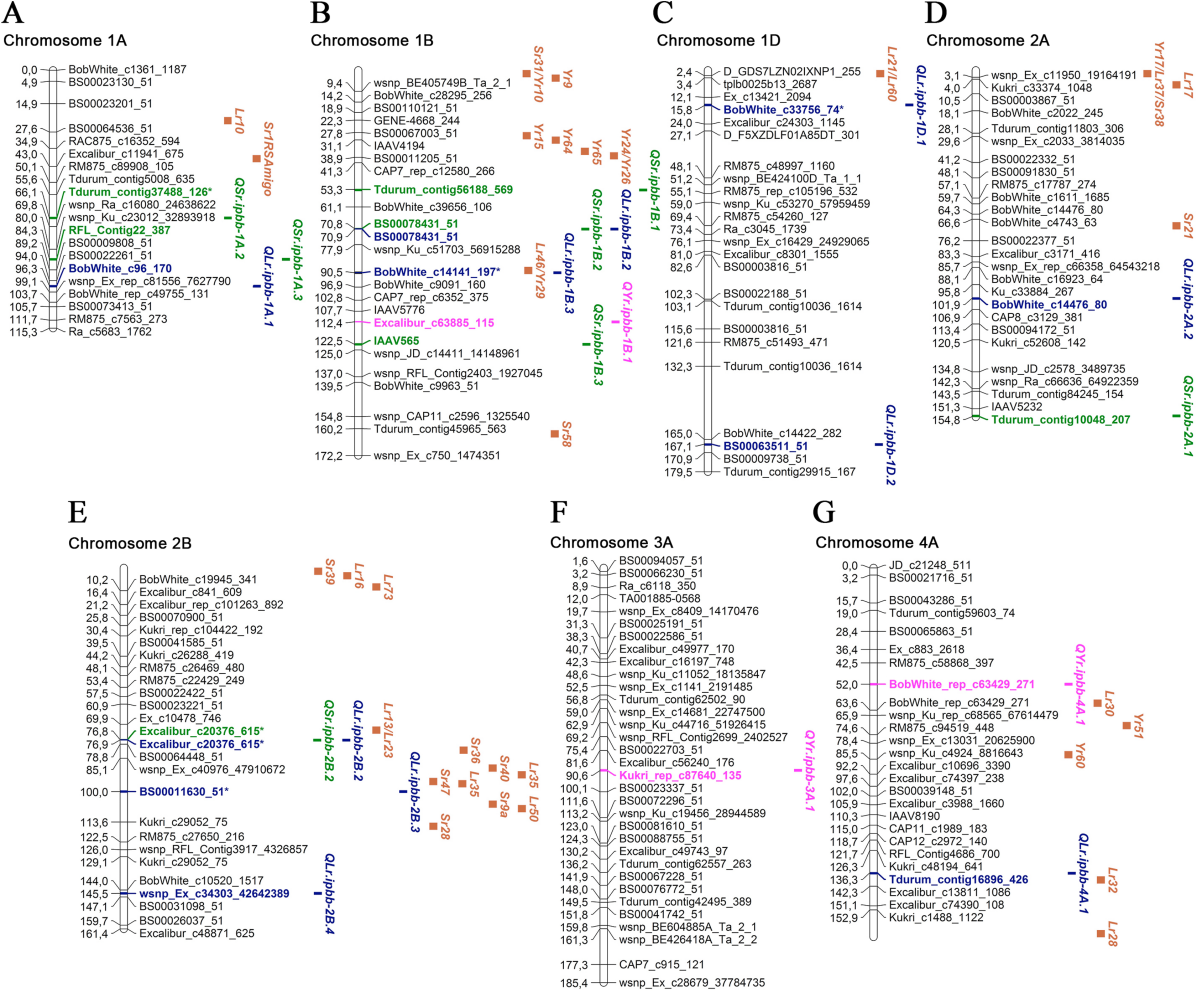
Genetic map of QTLs associated with resistance to leaf, stem, and yellow rusts and some possible candidate resistance genes. The SNP names are shown on the right and positions of marker loci are shown on the left of the linkage maps in centimorgans (cM). Significant markers, the QTLs identified in this study, and potential candidate resistance genes are highlighted in brown for genes, blue for LR QTLs, green for SR QTLs, and pink for YR QTLs. (A) Chromosome 1A; (B) Chromosome 1B; (C) Chromosome 1D; (D) Chromosome 2A; (E) Chromosome 2B; (F) Chromosome 3A; (G) Chromosome 4A.

**Figure 4:**
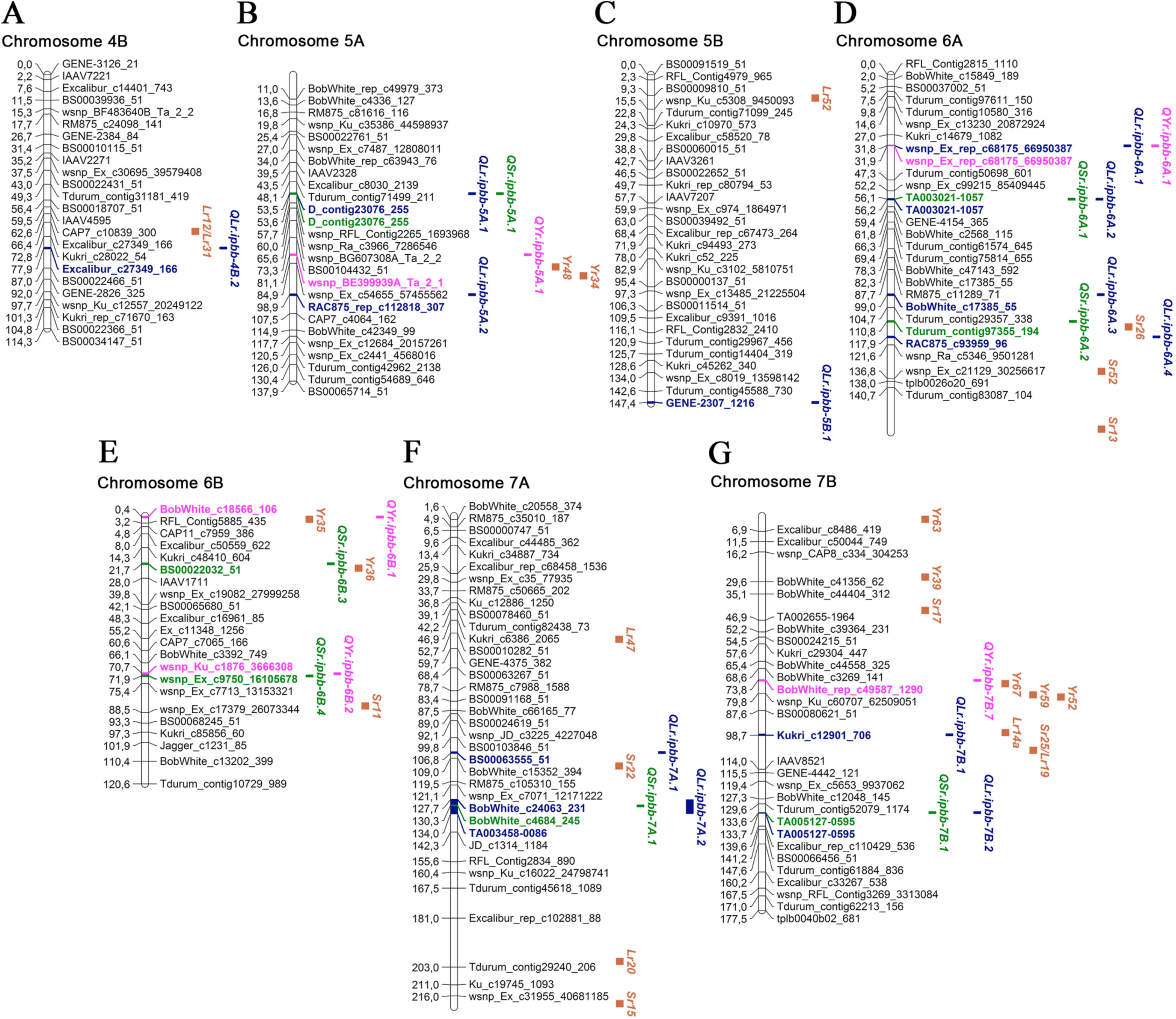
Genetic map of QTLs associated with resistance to leaf, stem, and yellow rusts and some possible candidate resistance genes. The SNP names are shown on the right and positions of marker loci are shown on the left of the linkage maps in centimorgans (cM). Significant markers, the identified QTLs, and potential candidate resistance genes from the literature are highlighted in brown for genes, blue for LR QTLs, green for SR QTLs, and pink for YR QTLs. (A) Chromosome 4B; (B) Chromosome 5A; (C) Chromosome 5B; (D) Chromosome 6A; (E) Chromosome 6B; (F) Chromosome 7A; (G) Chromosome 7B.

Overall, 45 MTAs with significant *P*-values were identified for LR, SR, and YR in 2018 and 2019. Manhattan and QQ plots of the GWAS results for all environments are provided in Table S2. All MTAs were designated as QTLs and positioned on a genetic map together with approximate positions of potential candidate *Lr*, *Sr*, and *Yr* genes (Table 3; Figs. 3 and 4; Table S3).

**Table 3:** The quantitative trait loci (QTLs) for resistance to leaf, stem, and yellow rusts.

#	Tr.	Marker	QTL	Chr.	Pos. (cM)^1^	2018				2019			
						*P*-value	Al.	Effect	R2	*P*-value	Al.	Effect	R2
1	LR	BobWhite_c96_170	*QLr.ipbb-1A.1*	1A	96.3	2.47E−04	A	−2.03	0.07	–	–	–	–
2	LR	BS00078431_51	*QLr.ipbb-1B.2*	1B	70.8	5.52E−12	C	3.56	0.25	4.01E−09	C	3.22	0.19
3	LR	BobWhite_c14141_197	*QLr.ipbb-1B.3*	1BL^2^	90.5^2^	4.70E−04	A	−2.12	0.06	8.41E−06	A	−2.83	0.12
4	LR	BobWhite_c33756_74	*QLr.ipbb-1D.1*	1DS^2^	15.8^2^	–	–	–	–	3.11E−04	G	1.77	0.07
5	LR	BS00063511_51	*QLr.ipbb-1D.2*	1D	167.1	3.06E−10	A	3.24	0.21	6.00E−07	A	2.64	0.14
6	LR	BobWhite_c14476_80	*QLr.ipbb-2A.2*	2A	101.9	–	–	–	–	1.19E−09	A	−3.38	0.21
7	LR	Excalibur_c20376_615	*QLr.ipbb-2B.2*	2BS^2^	76.9^2^	2.61E−14	C	4.28	0.33	3.29E−11	C	3.81	0.26
8	LR	BS00011630_51	*QLr.ipbb-2B.3*	2BL^2^	100.0^2^	–	–	–	–	2.32E−04	C	3.18	0.07
9	LR	wsnp_Ex_c34303_42642389	*QLr.ipbb-2B.4*	2B	145.5	6.82E−06	G	2.27	0.10	3.53E−05	G	2.19	0.09
10	LR	Tdurum_contig16896_426	*QLr.ipbb-4A.1*	4A	136.3	3.48E−04	C	−2.42	0.06	–	–	–	–
11	LR	Excalibur_c27349_166	*QLr.ipbb-4B.2*	4B	77.9	3.67E−10	C	−3.64	0.23	2.91E−06	C	−2.73	0.12
12	LR	D_contig23076_255	*QLr.ipbb-5A.1*	5A	53.5	3.09E−04	G	−2.01	0.09	–	–	–	–
13	LR	RAC875_rep_c112818_307	*QLr.ipbb-5A.2*	5A	98.9	4.72E−13	A	−4.01	0.28	5.86E−10	A	−3.52	0.22
14	LR	GENE-2307_1216	*QLr.ipbb-5B.1*	5B	147.4	5.72E−07	G	2.77	0.13	9.54E−06	G	2.58	0.11
15	LR	wsnp_Ex_rep_c68175_66950387	*QLr.ipbb-6A.1*	6A	31.9	3.64E−07	C	2.75	0.13	9.32E−07	C	2.79	0.13
16	LR	TA003021-1057	*QLr.ipbb-6A.2*	6A	56.1	9.13E−10	A	−3.41	0.19	9.84E−09	A	−3.30	0.18
17	LR	BobWhite_c17385_55	*QLr.ipbb-6A.3*	6A	99.0	8.89E−06	C	2.76	0.10	4.83E−05	C	2.66	0.09
18	LR	RAC875_c93959_96	*QLr.ipbb-6A.4*	6A	117.9	5.15E−05	A	−2.38	0.08	–	–	–	–
19	LR	BS00063555_51	*QLr.ipbb-7A.1*	7A	106.8	7.40E−08	C	3.21	0.15	2.18E−06	C	2.92	0.12
20	LR	BobWhite_c24063_231	*QLr.ipbb-7A.2*	7A	127.7	7.77E−10	C	3.34	0.20	7.95E−08	C	3.00	0.16
21	LR	TA003458-0086		7A	134.0	2.57E−07	C	2.89	0.14	3.15E−05	C	2.43	0.10
22	LR	Kukri_c12901_706	*QLr.ipbb-7B.1*	7B	98.7	–	–	–	–	3.55E−04	A	−2.33	0.07
23	LR	TA005127-0595	*QLr.ipbb-7B.2*	7B	133.6	1.24E−04	A	−2.53	0.07	7.04E−05	A	−2.74	0.09
24	SR	Tdurum_contig37488_126	*QSr.ipbb-1A.2*	1AS^2^	66.1^2^	–	–	–	–	4.35E−04	C	−3.08	0.07
25	SR	RFL_Contig22_387	*QSr.ipbb-1A.3*	1A	84.3	2.50E−04	G	−2.56	0.07	–	–	–	–
26	SR	Tdurum_contig56188_569	*QSr.ipbb-1B.1*	1B	53.3	–	–	–	–	9.36E−05	C	−3.15	0.08
27	SR	BS00078431_51	*QSr.ipbb-1B.2*	1B	70.8	–	–	–	–	3.48E−04	C	1.68	0.07
28	SR	IAAV565	*QSr.ipbb-1B.3*	1B	122.5	3.74E−04	A	−0.70	0.06	–	–	–	–
29	SR	Tdurum_contig10048_207	*QSr.ipbb-2A.1*	2A	154.8	4.41E−04	A	−2.56	0.06	–	–	–	–
30	SR	Excalibur_c20376_615	*QSr.ipbb-2B.2*	2BS^2^	76.9^2^	–	–	–	–	2.37E−05	C	2.08	0.10
31	SR	D_contig23076_255	*QSr.ipbb-5A.1*	5A	53.5	–	–	–	–	1.73E−04	A	−2.77	0.08
32	SR	TA003021-1057	*QSr.ipbb-6A.1*	6A	56.1	–	–	–	–	3.11E−04	A	−1.78	0.07
33	SR	Tdurum_contig97355_194	*QSr.ipbb-6A.2*	6A	110.8	4.58E−04	A	−2.42	0.06	–	–	–	–
34	SR	BS00022032_51	*QSr.ipbb-6B.3*	6B	21.7	2.03E−04	A	2.57	0.07	–	–	–	–
35	SR	wsnp_Ex_c9750_16105678	*QSr.ipbb-6B.4*	6B	71.9	–	–	–	–	3.67E−04	A	−1.94	0.07
36	SR	BobWhite_c4684_245	*QSr.ipbb-7A.1*	7A	130.3	1.83E−04	C	4.71	0.07	–	–	–	–
37	SR	TA005127-0595	*QSr.ipbb-7B.1*	7B	133.6	1.78E−04	A	2.59	0.07	–	–	–	–
38	YR	Excalibur_c63885_115	*QYr.ipbb-1B.1*	1B	112.4	–	–	–	–	2.84E−04	A	−1.20	0.06
39	YR	Kukri_rep_c87640_135	*QYr.ipbb-3A.1*	3A	90.6	2.01E−04	G	−1.50	0.06	–	–	–	–
40	YR	BobWhite_rep_c63429_271	*QYr.ipbb-4A.1*	4A	52.0	1.91E−04	A	1.88	0.06	–	–	–	–
41	YR	wsnp_BE399939A_Ta_2_1	*QYr.ipbb-5A.1*	5A	81.1	–	–	–	–	1.12E−04	C	−1.42	0.10
42	YR	wsnp_Ex_rep_c68175_66950387	*QYr.ipbb-6A.1*	6A	31.9	3.78E−04	C	−1.40	0.06	–	–	–	–
43	YR	BobWhite_c18566_106	*QYr.ipbb-6B.1*	6B	0.4	3.55E−04	A	1.75	0.06	–	–	–	–
44	YR	wsnp_Ku_c1876_3666308	*QYr.ipbb-6B.2*	6B	70.7	–	–	–	–	1.22E−04	C	1.59	0.07
45	YR	BobWhite_rep_c49587_1290	*QYr.ipbb-7B.1*	7B	73.8	3.14E−04	C	−1.46	0.06	–	–	–	–

**Notes:**

1 Positions according to 90K Array Consensus map.

2 Positions according to the CSS POPSEQ 2014 map.

Tr., trait; Al., effective allele; R2, Phenotypic variation explained by the QTL; LR, leaf rust; SR, stem rust; YR, yellow rust.

Five SNP markers were found to be associated with both LR and SR resistance: BS00078431_51, Excalibur_c20376_615, D_contig23076_255, TA003021-1057, and TA005127-0595 (Table 3). One SNP marker (wsnp_Ex_rep_c68175_66950387) was associated with resistance to LR and YR (Table 3). Twenty-three MTAs for LR resistance were identified on 12 chromosomes (Table 3; Figs. 3 and 4), explaining 6.2–33.0% of the variations in LR resistance in the studied collection. Fifteen of those MTAs were identified in both 2018 and 2019, while four MTAs were discovered in only 2018 and four MTAs in only 2019. For the six MTAs for LR resistance, the following possible candidate genes were identified: *Lr46* on 1B; *Lr21* and *Lr60* on 1D; *Lr13*, *Lr23*, *Lr35*, and *Lr50* on 2B; *Lr32* on 4A; *Lr12* and *Lr31* on 4B; and *Lr14* and *Lr19* on 7B (Figs. 3 and 4; Table S3). Comparative analysis of significant SNP sequences with the *T. aestivum* genome from the EnsemblPlants database revealed 20 protein-coding genes overlapping the MTAs for LR resistance identified in this study (Table S3), 15 of which are positioned in exon regions, and for three of these, information is available on their coded proteins. These three proteins are folate gamma-glutamyl hydrolase, growth-regulating factor 5-4A, and CNNM transmembrane domain-containing protein. Ten orthologous genes of *Aegilops tauschii* Coss., *Brachypodium distachyon* (L.) P. Beauv. and *Triticum urartu* Thumanjan ex Gandilyan were detected as overlapping with the MTAs for LR resistance identified in the current study (Table S3). The list includes ABC transporter A family member 2, tRNA pseudouridine synthase B, trimethylguanosine synthase, zinc transporter 7, tubby-like F-box protein, ETO1-like protein 1, putative membrane protein, 25.3 kDa vesicle transport protein, chitinase-like protein 1, and isoleucyl-tRNA synthetase.

For SR resistance, 14 MTAs were identified on nine chromosomes (1A, 1B, 2A, 2B, 5A, 6A, 6B, 7A, and 7B). Seven SR MTAs were identified based on field trials in 2018 and another seven in 2019, with no MTAs identified in both years (Table 3). In total, each of these MTAs explained up to 9.7% of the observed phenotypic variations. For three SR MTAs, the possible candidate genes were *Sr36*, *Sr40*, *Sr47*, *Sr9*, and *Sr28* on 2B; *Sr26* on 6A; and *Sr11* on 6B (Figs. 3 and 4; Table S3). The sequence alignment of SNP markers for the identified MTAs using the EnsemblPlants genome database suggested that nine QTLs for SR resistance were positioned in the exon regions of genes coding for uncharacterized proteins of *T. aestivum*. Three of them have orthologous genes in related species (Table S3), which code for Ras-related protein Rab11B, allantoinase enzyme, and tubby-like F-box protein of *A. tauschii*, *T. urartu*, and *B. distachyon*, respectively.

In the YR study, eight MTAs were identified on seven chromosomes, and each of them explained 5.6–10.2% of the total variation in YR resistance (Table 3; Figs. 3 and 4). Five and three YR resistance MTAs were detected in 2018 and 2019, respectively. Three MTAs had several possible candidate genes: *Yr34* and *Yr48* on 5A; *Yr35* on 6B; and *Yr52*, *Yr59*, and *Yr67* on 7B (Figs. 3 and 4; Table S3). The identified SNP markers in five of eight QTLs for YR resistance were in genes coding for uncharacterized proteins, with one gene coding for a tubulin α chain protein in *T. aestivum* (Table S3). Three genes had orthologues in *A. tauschii*: ribosomal L1 domain-containing protein 1, 25.3 kDa vesicle transport protein, and E3 ubiquitin-protein ligase makorin. One gene shared high identity with the gene coding for eukaryotic elongation factor 1 gamma of *Hordeum vulgare* L. (Table S3).

## Discussion

### Resistance of studied accessions in the field

Among the studied wheat accessions, six lines from Kazakhstan (IR-38, E-736, E-756, E-795, E-819, and E-607) were resistant to all three diseases in 2018. In 2019, only three of these (E-736, E-746, and E-607) were resistant to LR and SR and moderately resistant to YR. Fourteen accessions, including nine promising breeding lines from Kazakhstan (GWK 2161, E-736, E-746, E-756, E-757, E-761, E-806, E-815, and E-607), three from Russia (lutescens 1300, lutescens 151-03-85, and lutescens 242-97-2-26), one cultivar from Kazakhstan (“Pavlodarskaya Yubileinaya”), and one cultivar from Russia (“Silach”) were stably immune to LR in both 2018 and 2019. Eleven wheat accessions demonstrated stable resistance to SR in both years. Among those accessions, there were six breeding lines from Kazakhstan (IR-38, E-607, E-736, E-746, E-760, and E-795), two cultivars from the USA (“Gatcher” and “Agent”), two cultivars (“Pavon 76” and “Buckbuck”) from Mexico, and “Seri 82” from Australia. As for resistance to YR, 13 accessions were immune in the 2 years. This group included one hybrid line 6,569 × *Triticum millitinae* Zhuk. from Kazakhstan and 12 European cultivars. Thus, all of the above-listed cultivars and lines from Kazakhstan and other countries are promising sources for breeding for rust resistance in wheat.

The presence of strong positive correlations among the resistance to the three studied diseases (Table 1) indicates the presence of possible pleiotropic genetic factors providing resistance to multiple pathogen species. This was confirmed by the presence of five MTAs associated with resistance to two diseases (Table 3). A strong positive correlation was observed between the 2 years (Table 1) which, together with ANOVA results, high heritability values (Table 2), and multiple occurrences of the same MTAs in 2 years (Table 3), indicated the stability of the genetic factors identified in this study.

### MTAs for LR resistance

Of 23 MTAs for LR resistance, 15 were detected in both 2018 and 2019, confirming the potential stable genetic role of their association to disease resistance. Of the 23 identified QTL, seven were located close to well-known mapped *Lr* genes (Table 3; Figs. 3 and 4; Table S3). We found *QLr.ipbb-2B.2* (Excalibur_c20376_615) were also associated with SR resistance. The results suggest that *QLr.ipbb-4B.2* (Excalibur_c27349_166) and *QLr.ipbb-7B.1* (Kukri_c12901_706) are located closely to genes *Lr12/Lr31* and *Lr14*/*Lr19*, respectively (Fig. 4). Genes *Lr12* and *Lr19* were previously shown to be highly effective against LR in southeast Kazakhstan (Koyshybaev, 2018). Other QTLs positioned in proximity to several *Lr* genes were *QLr.ipbb-1B.3* (BobWhite_c14141_197) near *Lr46*, *QLr.ipbb-1D.1* (BobWhite_c33756_74) close to *Lr21/Lr60*, and *QLr.ipbb-4A.1* (Tdurum_contig16896_426) in the vicinity of *Lr32* (Fig. 3; Table S3). However, these genes are only potential candidates for identified MTAs because the process of genes identification is heavily reliant on the markers used in the mapping. The remaining 16 MTAs were presumably positioned a significant distance from *Lr* genes on corresponding chromosomes. However, 13 of them have similar regions associated with LR resistance genes identified in previous research. For example, GWAS in durum wheat (Aoun et al., 2016) revealed six MTAs of LR resistance overlapping or located close to the following loci identified in this study: *QLr.ipbb-1A.1* (BobWhite_c96_170), *QLr.ipbb-2A.2* (BobWhite_c14476_80), *QLr.ipbb-2B.4* (wsnp_Ex_c34303_42642389), *QLr.ipbb-5A.2* (RAC875_rep_c112818_307), *QLr.ipbb-7A.2* (BobWhite_c24063_231 and TA003458-0086), and *QLr.ipbb-6A.4* (RAC875_c93959_96). Loci *QLr.ipbb-5A.1* (D_contig23076_255), *QLr.ipbb-6A.1* (wsnp_Ex_rep_c68175_66950387), and *QLr.ipbb-6A.3* (BobWhite_c17385_55) from this study were in regions close to *QLr.uaf.5AS.2*, *QLr.uaf.6AS.2*, and *QLr.uaf.6AL.1* which were identified by Muhammad et al. (2018). One of the QTLs similar to *QLr.ipbb-6A.2* (TA003021-1057) was earlier described by Gao et al. (2016) as a genetic factor responsible for LR resistance. The QTL *QLr.ipbb-5B.1* (GENE-2307_1216 at 147.4 cM) is possibly located within the interval 140.1–156.4 cM of *QLr.fcu-5BL* discovered by Chu et al. (2009). Notably, *QLr.ipbb-1D.2* (BS00063511_51), *QLr.ipbb-7A.1* (BS00063555_51), and *QLr.ipbb-7B.2* (TA005127-0595) correspond to chromosomal locations that have not previously been detected in LR resistance studies; therefore, they represent potentially novel QTLs for LR resistance.

### MTAs for SR resistance

Based on the information from the literature survey, all MTAs of SR resistance identified in the current study can be subdivided into three categories: (1) MTAs that have mapped *Sr* candidate genes, (2) MTAs corresponding to QTLs from previous studies, and (3) novel MTAs detected in this work. The first group includes four MTAs positioned closely to *Sr* genes. The QTL *QSr.ipbb-2B.2* (Excalibur_c20376_615), located at 76.8 cM of the 2B chromosome, is close to a cluster of resistance genes, including several *Lr* genes together with *Sr36*, *Sr40*, *Sr47*, *Sr9*, and *Sr28* (Fig. 3; Table S3). *Sr36* and *Sr40* derived from *Triticum timopheevii* Zhuk. and *Sr47* transferred from *Aegilops speltoides* Tausch. are important genes providing resistance to the Ug99 (TTKSK) race of *P. graminis* f. sp. *tritici* (Faris et al., 2008; Wu, Pumphrey & Bai, 2009; Chemayek et al., 2016). *Sr28* was also reported to be effective against Ug99 (Rouse et al., 2012). The second QTL was *QSr.ipbb-6A.2* (Tdurum_contig97355_194) on chromosome 6A, located closely to *Sr26* (Fig. 4). *Sr26* is one of a few known major resistance genes effective against the Ug99 race (TTKSK) and its derivative TTKST (Liu et al., 2010). The next two QTLs, *QSr.ipbb-6B.4* (wsnp_Ex_c9750_16105678) and *QSr.ipbb-7A.1* (BobWhite_c4684_245), are located relatively close to genes *Sr11* and *Sr22*, respectively. The population of *P. graminis* f. sp. *tritici* in Kazakhstan is represented by six pathotypes: TFK/R, TDT/H, TTH/K, TKT/C, TKH/R, and TPS/H (Rsaliyev & Rsaliyev, 2018). Therefore, *Sr11* and *Sr36* are possibly effective against the majority of SR pathogen populations found in Kazakhstan.

The second group comprises nine QTLs located in similar genetic regions that have previously been described to be responsible for SR resistance. QTLs *QSr.ipbb-1A.2* (Tdurum_contig37488_126) at 66.1 cM and *QSr.ipbb-1A.3* (RFL_Contig22_387) at 84.3 cM are similar to the QTL mentioned in Elbasyoni et al. (2017) at 57.6 and 85.0 cM, respectively. Three QTLs on chromosome 3B (*QSr.ipbb-1B.1* (Tdurum_contig56188_569) at 53.3 cM, *QSr.ipbb-1B.2* (BS00078431_51) at 70.8 cM, and *QSr.ipbb-1B.3* (IAAV565) at 122.5 cM) were similar to those reported in other works at 61.4 cM (Elbasyoni et al., 2017), 74.4 cM (Muleta et al., 2017), and 119.9 cM (Elbasyoni et al., 2017), respectively. The *QSr.ipbb-2A.1* (Tdurum_contig10048_207) at 154.8 cM is close to the two QTLs identified in a nested association mapping study at 144 and 145 cM (Bajgain et al., 2016). QTL *QSr.ipbb-5A.1* (D_contig23076_255), located at 53.5 cM on chromosome 5A, is similar to wPt-5588 (Prins et al., 2016). The *QSr.ipbb-6A.1* (TA003021-1057) at 56.1 cM on chromosome 6A is close to the genetic factor previously described for SR resistance at 63.2 cM (Elbasyoni et al., 2017). The comparative assessment of the *QSr.ipbb-7B.1* (TA005127-0595), located at 133.6 cM on the long arm of chromosome 7B, showed no similarity with known SR resistance genes suggesting the novelty of this QTL.

### MTAs for YR resistance

Of the eight MTAs for YR resistance identified in this study, three were located close to mapped *Yr* genes (Figs. 3 and 4; Table S3). One of them is *QYr.ipbb-5A.1* (RAC875_rep_c112818_307), positioned at 81.1 cM on chromosome 5A, closely to *Yr48* and *Yr34*. The next QTL is *QYr.ipbb-6B.1* (BobWhite_c18566_106), located on the short arm of chromosome 6B, as well as the seedling resistance gene *Yr35* transferred from *Triticum dicoccoides* Schrank ex Schübl. (Marais et al., 2005). The SNP BobWhite_rep_c49587_1290 of the QTL *QYr.ipbb-7B.7* is positioned at 73.8 cM on chromosome 7B, which is close to the cluster of genes *Yr67*, *Yr59*, and *Yr52*. However, none of these genes were previously identified as effective in the southern Kazakhstan (Koyshybaev, 2018).

The remaining five MTAs revealed in this study coincide with YR resistance loci identified in previous studies. For instance, QTL *QYr.ipbb-1B.1* (Excalibur_c63885_115), located at 112.4 cM on chromosome 1B is close to SNP IWA1825 (109.06 cM). This SNP was reported to be associated with YR resistance by Maccaferri et al. (2015). The *QYr.ipbb-3A.1* (Kukri_rep_c87640_135) at 90.6 cM is genetically close to IWA2332 at 102.09 cM (Maccaferri et al., 2015). QTLs *QYr.ipbb-4A.1* and *QYr.ipbb-6A.1* were similar to YR resistance genes/QTLs located on chromosomes 4A and 6A, respectively (Yao et al., 2019*)*, whereas *QYr.ipbb-6B.2* at chromosome 6B was close to the QTL reported by Godoy et al. (2018).

### Comparison of MTAs with protein-coding genes in the reference genome of *T. aestivum* and related species

Among the protein-coding genes overlapping MTAs for LR resistance identified in this study (Table S3), three may be involved in resistance to rust pathogens. One of those genes codes for chitinase-like protein 1 and overlaps *QLr.ipbb-7A.2*. Chitinase is an antifungal hydrolase that is important in the wheat defense response to SR (Münch-Garthoff et al., 1997), YR (Huang et al., 2013), and LR (Anguelova-Merhar, Westhuizen & Pretorius, 2008). The second gene codes zinc transporter 7 in *A. tauschii*, which is genetically positioned close to the gene overlapping *QLr.ipbb-2A.2*. According to the elemental defense hypothesis, the accumulation of trace elements in plants may be similar to organic defenses, and the joint effect hypothesis proposes that trace elements and organic defenses can be effective against pathogens or herbivores (Martos et al., 2016). Another important protein is ETO1-like protein 1 of *A. tauschii*, which is involved in ethylene biosynthesis. Given its ability to easily permeate through cell membranes, ethylene provides a mechanism for plants to rapidly coordinate their response to adverse environments, such as pathogen attack (Christians et al., 2009). The gene encoding this protein is orthologous to *QLr.ipbb-2B.4* identified in this study. The remaining proteins have not been reported to be directly involved in the resistance of wheat to fungal diseases, but might still be involved in the defense process. In particular, membrane proteins such as ABC transporter A family member 2 and putative membrane protein of *A. tauschii* are similar to the genes of *T. aestivum* overlapping *QLr.ipbb-1A.1* and *QLr.ipbb-4B.2*, respectively (Table S3).

The search for genes overlapping the identified QTLs for SR resistance in this study revealed three similar orthologue genes from other plant species (Table S3) that may possibly be involved in plant defense against pathogens. The first example is the SNP marker IAAV565 underlying *QSr.ipbb-1B.3*, which overlaps the gene of *A. tauschii* coding for the Ras-related protein Rab11B—one of the key regulators of membrane traffic (Asaoka et al., 2013). The second is the *QSr.ipbb-2A.1* (Tdurum_contig10048_207), which is in the exon of the gene that codes for the allantoinase enzyme in *T. urartu*. This enzyme plays an important role in the assimilation, metabolism, transport, and storage of nitrogen in plants (Yang & Han, 2004). The third was *QSr.ipbb-2B.2*. Like *QLr.ipbb-2B.2*, which is located in the exon of the gene whose orthologue in *B. distachyon* coding for tubby-like F-box protein. F-box proteins regulate many different cellular processes, such as cell cycle transition, transcriptional regulation, and signal transduction (Lai et al., 2004; Bao et al., 2014).

SNP BobWhite_rep_c63429_271 located on chromosome 4A and associated with YR resistance overlaps the gene coding for tubulin α chain in *T. aestivum* (Table S3). Tubulin is the protein subunit of microtubules, which are responsible for intracellular transport, cell division, and eukaryotic cell shape in plants (Hashimoto, 2015).

However, all MTAs identified in this study are hypothetical candidate loci for resistance to a particular type of wheat rusts. Each of the associations should be studied further to confirm their actual roles in resistance to pathogen(s). The overlap of SNP markers and protein-coding genes of wheat or related species does not prove the role of these proteins in resistance to rusts, though it indicates their potential involvement in the complex process of plant resistance.

## Conclusions

In this study, 45 loci associated with the resistance to three rust diseases were identified in a collection of 215 common wheat accessions. The identified loci included 23 QTLs for resistance to LR, 14 QTLs for resistance to SR, and eight QTLs for resistance to YR. Among them, five loci were associated with both LR and SR resistance, and one locus was associated with resistance to LR and YR. Three QTLs for LR resistance, *QLr.ipbb-1D.2* (BS00063511_51), *QLr.ipbb-7A.1* (BS00063555_51), and *QLr.ipbb-7B.2* (TA005127-0595), and one QTL for SR resistance, *QSr.ipbb-7B.1* (TA005127-0595), are likely novel. Comparative analysis of the QTLs revealed six candidate protein-coding genes previously characterized as regulatory genes related to pathogenesis.

## Supplemental Information

10.7717/peerj.9820/supp-1 Supplemental Information 1 The raw data for resistance in the collection of 215 accessions to leaf, stem, and yellow rust diseases.

10.7717/peerj.9820/supp-2 Supplemental Information 2 HapMap is containg 12419 SNP marker for 215 accessions of common wheat.

10.7717/peerj.9820/supp-3 Supplemental Information 3 List of common wheat cultivars and breeding lines used in the currient study.

10.7717/peerj.9820/supp-4 Supplemental Information 4 Manhattan and QQ plots with MTAs for LR, SR, and YR identified in the current study. Positions of SNPs are given according to 20K Illumina iSelect SNP assay (cM) and multiplied on 10 in order to fit TASSEL requerments. Red line denotes selected threshold at *P* < 1E-3

10.7717/peerj.9820/supp-5 Supplemental Information 5 The list of possible candidate *Lr*, *Sr* and *Yr* genes, protein-coding genes of *T. aestivum* overlapping with QTL identified in the current study and proteins coded by them. Orthologue genes and proteins with known functions in other species are listed for proteins whose functions are uncharacterized in *T. aestivum*.

## Additional Information and Declarations

### Data Availability

The following information was supplied regarding data availability: Raw data is available in the Supplemental Files.
