# Supplementary material for: Genome-wide association mapping for resistance to leaf, stem, and yellow rusts of common wheat under field conditions of South Kazakhstan
Source: PeerJ. 2020 Aug 31;8:e9820. doi: 10.7717/peerj.9820 (PMC7469934; doi:10.7717/peerj.9820)
Supplement: Supplemental Information 5 — Orthologue genes and proteins with known functions in other species are listed for proteins whose functions are uncharacterized in T. aestivum. [file peerj-08-9820-s005.docx]

**Table S3. The list of possible candidate *Lr*, *Sr* and *Yr* genes, protein-coding genes of *T. aestivum* overlapping with QTL identified in the current study and proteins coded by them.** Orthologue genes and proteins with known functions in other species are listed for proteins whose functions are uncharacterized in *T. aestivum.*

| **#** | **Trait** | **Marker** | **Chr.^1^** | **Pos. (cM)^1^** | **Candidate gene(s)** | **Overlapping gene(s)** | **Protein** | **Orthologue gene(s)^3^** | **Identity (%)^4^** | **Orthologue protein^3^** |
| --- | --- | --- | --- | --- | --- | --- | --- | --- | --- | --- |
| 1 | LR | BobWhite_c96_170 | 1A | 96.3 |  | TraesCS1A02G342900 (exon) | Uncharacterized protein | AET1Gv20821000  (*A. tauschii*) | 97.72 | ABC transporter A family member 2 |
| 2 | LR | BS00078431_51 | 1B | 70.8 |  | TraesCS1B02G266000 (intron) | Uncharacterized protein | - | - | - |
| 3 | LR | BobWhite_c14141_197 | 1BL^2^ | 90.5^2^ | *Lr46* | TraesCS1B02G350700 (exon) | Folate gamma-glutamyl hydrolase | - | - | - |
| 4 | LR | BobWhite_c33756_74 | 1DS^2^ | 15.8^2^ | *Lr21, Lr60* | TraesCS1D02G024800 (exon) | Uncharacterized protein | AET1Gv20053700  (*A. tauschii*) | 98.03 | tRNA pseudouridine synthase B |
| 5 | LR | BS00063511_51 | 1D | 167.1 |  | TraesCS1D02G439800 (intron) | Uncharacterized protein | AET1Gv21018700  (*A. tauschii*) | 99.32 | Trimethylguanosine synthase |
| 6 | LR | BobWhite_c14476_80 | 2A | 101.9 |  | TraesCS2A02G141400 (exon) | Uncharacterized protein | AET2Gv20287000  (*A. tauschii*) | 96.29 | Zinc transporter 7 |
| 7 | LR | Excalibur_c20376_615 | 2BS^2^ | 76.9^2^ | *Lr13, Lr23* | TraesCS2B02G141800 (exon) | Uncharacterized protein | BRADI_1g18380v3  (*B. distachyon*) | 79.63 | Tubby-like F-box protein |
| 8 | LR | BS00011630_51 | 2BL^2^ | 100.0^2^ | *Lr35, Lr50* | TraesCS2B02G490800 (exon) | Uncharacterized protein | - | - | - |
| 9 | LR | wsnp_Ex_c34303_42642389 | 2B | 145.5 |  | TraesCS2B02G592500 (exon) | Uncharacterized protein | AET2Gv21232800  (*A. tauschii*) | 99.44 | ETO1-like protein 1 |
| 10 | LR | Tdurum_contig16896_426 | 4A | 136.3 | *Lr32* | TraesCS4A02G434900 (exon) | Growth regulating factor 5-4A | - | - | - |
| 11 | LR | Excalibur_c27349_166 | 4B | 77.9 | *Lr12, Lr31* | TraesCS4B02G328500 (exon) | Uncharacterized protein | AET4Gv20774800  (*A. tauschii*) | 100 | Putative membrane protein |
| 12 | LR | D_contig23076_255 | 5A | 53.5 |  | TraesCS5A02G264000 (exon/intron) | Uncharacterized protein | - | - | - |
| 13 | LR | RAC875_rep_c112818_307 | 5A | 98.9 |  | TraesCS5A02G428800 (exon) | Uncharacterized protein | AET5Gv20995500  (*A. tauschii*) | 100 | 25.3 kDa vesicle transport protein |
| 14 | LR | GENE-2307_1216 | 5B | 147.4 |  | TraesCS5B02G505200 (intron) | Uncharacterized protein | - | - | - |
| 15 | LR | wsnp_Ex_rep_c68175_66950387 | 6A | 31.9 |  | - | - | - | - | - |
| 16 | LR | TA003021-1057 | 6A | 56.1 |  | - | - | - | - | - |
| 17 | LR | BobWhite_c17385_55 | 6A | 99 |  | TraesCS6A02G349000 (exon) | Uncharacterized protein | - | - | - |
| 18 | LR | RAC875_c93959_96 | 6A | 117.9 |  | TraesCS6A02G369300 (exon) | Uncharacterized protein | - | - | - |
| 19 | LR | BS00063555_51 | 7A | 106.8 |  | - | - | - | - | - |
| 20 | LR | BobWhite_c24063_231 | 7A | 127.7 |  | TraesCS7A02G250500 (exon) | Uncharacterized protein | TRIUR3_01974  (*T. urartu*) | 98.18 | Chitinase-like protein 1 |
| 21 | LR | TA003458-0086 | 7A | 134 |  | TraesCS7A02G389100 (intron) | Uncharacterized protein | - | - | - |
| 22 | LR | Kukri_c12901_706 | 7B | 98.7 | *Lr14, Lr19* | TraesCS7B02G381300 (exon) | CNNM transmembrane domain-containing protein | - | - | - |
| 23 | LR | TA005127-0595 | 7B | 133.6 |  | TraesCS7B02G432400 (exon) | Uncharacterized protein | AET7Gv21263100  (*A. tauschii*) | 99.32 | Isoleucyl-tRNA synthetase |
| 24 | SR | Tdurum_contig37488_126 | 1AS^2^ | 66.1^2^ |  | TraesCS1A02G060500 (exon) | Uncharacterized protein | - | - | - |
| 25 | SR | RFL_Contig22_387 | 1A | 84.3 |  | TraesCS1A02G312000 (exon) | Uncharacterized protein | - | - | - |
| 26 | SR | Tdurum_contig56188_569 | 1B | 53.3 |  | TraesCS1B02G048800 (exon) | Uncharacterized protein | - | - | - |
| 27 | SR | BS00078431_51 | 1B | 70.8 |  | TraesCS1B02G266000 (intron) | Uncharacterized protein | - | - | - |
| 28 | SR | IAAV565 | 1B | 122.5 |  | TraesCS1B02G426300 (exon) | Uncharacterized protein | AET1Gv20952800  (*A. tauschii*) | 99.54 | Ras-related protein Rab11B |
| 29 | SR | Tdurum_contig10048_207 | 2A | 154.8 |  | TraesCS2A02G588000 (exon) | Uncharacterized protein | TRIUR3_06369  (*T. urartu*) | 80.13 | Allantoinase |
| 30 | SR | Excalibur_c20376_615 | 2BS^2^ | 76.9^2^ | *Sr36, Sr40, Sr47, Sr9, Sr28* | TraesCS2B02G141800 (exon) | Uncharacterized protein | BRADI_1g18380v3  (*B. distachyon*) | 79.63 | Tubby-like F-box protein |
| 31 | SR | D_contig23076_255 | 5A | 53.5 |  | TraesCS5A02G264000 (intron) | Uncharacterized protein | - | - | - |
| 32 | SR | TA003021-1057 | 6A | 56.1 |  | - | - | - | - | - |
| 33 | SR | Tdurum_contig97355_194 | 6A | 110.8 | *Sr26* | TraesCS6A02G360900 (exon) | Uncharacterized protein | - | - | - |
| 34 | SR | BS00022032_51 | 6B | 21.7 |  | - | - | - | - | - |
| 35 | SR | wsnp_Ex_c9750_16105678 | 6B | 71.9 | *Sr11* | TraesCS6B02G348700 (exon/intron) | Uncharacterized protein | - | - | - |
| 36 | SR | BobWhite_c4684_245 | 7A | 130.3 |  | - | - | - | - | - |
| 37 | SR | Excalibur_rep_c75066_126 | 7B | 133.6 |  | TraesCS7B02G431600 (exon) | Uncharacterized protein | - | - | - |
| 38 | YR | Excalibur_c63885_115 | 1B | 112.4 |  | TraesCS1B02G417200 (exon) | Uncharacterized protein | AET1Gv20935500  (*A. tauschii*) | 81.05 | Ribosomal L1 domain-containing protein 1 |
| 39 | YR | Kukri_rep_c87640_135 | 3A | 90.6 |  | TraesCS3A02G328300 (exon) | Uncharacterized protein | - | - | - |
| 40 | YR | BobWhite_rep_c63429_271 | 4A | 52 |  | TraesCS4A02G257800 (exon) | Tubulin alpha chain | - | - | - |
| 41 | YR | RAC875_rep_c112818_307 | 5A | 98.9 | *Yr34, Yr48* | TraesCS5A02G428800 (exon) | Uncharacterized protein | AET5Gv20995500  (*A. tauschii*) | 100 | 25.3 kDa vesicle transport protein |
| 42 | YR | wsnp_Ex_rep_c68175_66950387 | 6A | 31.9 |  | - | - | - | - | - |
| 43 | YR | BobWhite_c18566_106 | 6B | 0.4 | *Yr35* | - | - | - | - | - |
| 44 | YR | wsnp_Ku_c1876_3666308 | 6B | 70.7 |  | TraesCS6B02G310500 (exon) | Uncharacterized protein | AET6Gv20712000  (*A. tauschii*) | 82.88 | E3 ubiquitin-protein ligase makorin |
| 45 | YR | BobWhite_rep_c49587_1290 | 7B | 73.8 | *Yr52, Yr59, Yr67* | TraesCS7B02G266300 (exon) | Uncharacterized protein | HORVU7Hr1G087050 (*H. vulgare*) | 94.63 | Eukaryotic elongation factor 1 gamma |
| Note: ^1^ – Positions according to 90K array consensus map; ^2^ – Positions according to the CSS POPSEQ 2014 map; ^3^ – Genes and proteins with known functions only are included; ^4^ – Percentage of *T. aestivum’s* sequence matching the sequence of the orthologue. | | | | | | | | | | |
